# Mitochondrial DNA variants in a cohort from Argentina with suspected Leber’s hereditary optic neuropathy (LHON)

**DOI:** 10.1371/journal.pone.0275703

**Published:** 2023-02-24

**Authors:** Paula I. Buonfiglio, Sebastián Menazzi, Liliana Francipane, Vanesa Lotersztein, Verónica Ferreiro, Ana Belén Elgoyhen, Viviana Dalamón

**Affiliations:** 1 Laboratorio de Fisiología y Genética de la Audición, Instituto de Investigaciones en Ingeniería Genética y Biología Molecular “Dr. Héctor N. Torres”, Consejo Nacional de Investigaciones Científicas y Técnicas - INGEBI / CONICET, Ciudad Autónoma de Buenos Aires, Argentina; 2 División Genética, Hospital de Clínicas “José de San Martín”, Ciudad Autónoma de Buenos Aires, Argentina; 3 Servicio de Genética, Hospital Militar Central “Dr. Cosme Argerich”, Ciudad Autónoma de Buenos Aires, Argentina; 4 Laboratorio Genos, Ciudad Autónoma de Buenos Aires, Argentina; 5 Departamento de Farmacología, Facultad de Medicina, Universidad de Buenos Aires, C1121ABG, Ciudad Autónoma de Buenos Aires, Argentina; University of Parma, ITALY

## Abstract

The present study investigates the spectrum and analysis of mitochondrial DNA (mtDNA) variants associated with Leber hereditary optic neuropathy (LHON) in an Argentinean cohort, analyzing 3 LHON-associated mitochondrial genes. In 32% of the cases, molecular confirmation of the diagnosis could be established, due to the identification of disease-causing variants. A total of 54 variants were observed in a cohort of 100 patients tested with direct sequencing analysis. The frequent causative mutations m.11778G>A in *MT-ND*4, m.3460G>A in *MT-ND*1, and m.14484T>C in *MT-ND*6 were identified in 28% of the cases of our cohort. Secondary mutations in this Argentinean LHON cohort were m.11253T>C p.Ile165Thr in *MT-ND*4, identified in three patients (3/100, 3%) and m.3395A>G p.Tyr30Cys in *MT-ND*1, in one of the patients studied (1%). This study shows, for the first time, the analysis of mtDNA variants in patients with a probable diagnosis of LHON in Argentina. Standard molecular methods are an effective first approach in order to achieve genetic diagnosis of the disease, leaving NGS tests for those patients with negative results.

## Introduction

Leber hereditary optic neuropathy (LHON; OMIM 535000) is a neuro ophthalmologic mitochondrial disease associated with rapid, painless, acute or subacute bilateral visual loss in young adults, predominantly caused by mutations in mitochondrial DNA (mtDNA) [1, 2]. However, mutations in nuclear genes have been reported such as *DNAJC*30, *NDUFS*2 and *MCAT* for some unsolved LHON patients whereas *OPA*1, *TYMP* and *POLG* for other similar associated phenotypes [3, 4]. Additionally, in some patients with LHON other clinical symptoms, such as movement disorders, dystonia, and multiple-sclerosis-like illness are seen, which complicates the diagnosis in the clinical setting [5, 6]. The incidence of LHON ranges between 1:31,000 and 1:54.000, affecting predominantly males (in 80–90% of cases), with typical onset of symptoms between the 2nd and 3rd decades of life. In Argentina, high levels of underdiagnosis are likely, since molecular testing is not widely available and neuro ophthalmologists are scarce.

LHON is due to variants in the mitochondrial genome, which is a double-stranded 16,569-nucleotide pair circular molecule consisting of one D-Loop region and 37 genes. In particular, mutations in the mitochondrial genes encoding the NADH dehydrogenase (ND) subunits 1, 4, and 6 (*MT-ND*1, *MT-ND*4, and *MT-ND*6, respectively) are the most frequent causes of LHON, accounting for more than 90% of LHON diagnosis in a Caucasian population [7, 8]. The three most common causative mutations, described by several authors as the “primary variants” for the disease, are m.11778G>A in *MT‑ND*4 (by far the most common, accounting for 70% of all LHON cases worldwide), m.14484T>C in *MT‑ND*6 and m.3460G>A in *MT‑ND*1 [9]. According to Mitomap, more than 40 point mutations in mtDNA are associated with LHON, with an incidence that varies between different ethnic backgrounds [10].

NADH dehydrogenase subunits form part of the respiratory chain complex I. Alterations introduced by the primary LHON mutations contribute to decreased ATP and increased reactive oxygen species (ROS) production, leading to oxidative stress, which activates apoptosis in retinal ganglion cells, contributing to the pathophysiology of the disease [11]. The underlying physiopathology involves the dysfunction of the optic nerve caused by respiratory chain abnormalities, which tend to worsen over time [12–14]. The rapid and progressive loss of vision, usually with central visual scotoma, is directly related to the injury and death of retinal ganglion cells (RGC) [15].

Patients affected with LHON, mostly young men, usually experience sudden onset, painless, central vision loss which is frequently bilateral, or unilateral followed shortly (in average two months later) by contralateral affection. The peak age of onset in LHON is between 15 and 30 years, and 95% of carriers will experience visual failure before the age of 50 years [16]. Affected individuals are often aware of other affected family members, but up to 40% have no family history, mainly due to the emergence of *de novo* variants, variable penetrance and heteroplasmy [5, 17]. Being a mitochondrial disorder, family history is via maternal inheritance. Besides males who harbor pathogenic variants develop symptoms 5 times more often than females. Moreover, 50% of men and 90% of women with a primary LHON-causing mtDNA pathogenic variant do not develop blindness [5]. The visual prognosis in LHON is generally poor, and the majority of affected patients will end up being legally blind, with visual acuity of 20/200 or worse and a significant detrimental impact on their quality of life. Genotype-phenotype correlation has been established for some variants, which is relevant for the prognosis upon a molecular diagnosis [9, 16, 18]. Even though currently there is no curative treatment for this disorder, genetic counseling is paramount once the condition has been diagnosed, since measures to reduce the risk of affected offspring are available [19]. Idebenone has been approved to treat visual impairment, due to its antioxidant and mitochondrial electron carrier properties, preventing further vision loss. Vision recovery due to this treatment has been confirmed as well, both in randomized clinical trials and real-world data [20–23]. Additionally, ongoing clinical trials are evaluating the effects of intravitreal gene therapy for the treatment of ND4-LHON, and the results of some phase III trials have been successful [24–28]. Therefore, although treatment options remain limited, LHON research has now entered an active translational phase, with ongoing clinical trials to correct the underlying pathogenic mtDNA mutation.

Consequently, accurate molecular testing becomes crucial for establishing the diagnosis, precise genetic counseling and the immediate implementation of treatments. In this study, we aimed to identify the genetic variant distribution in a large Argentinian cohort of patients with a clinical diagnosis of LHON.

## Methods

### Patients

A total number of 100 non-related individuals (74 males, 26 females) with suspected LHON were sequentially referred to the Laboratory of Physiology and Genetics of Hearing, INGEBI/CONICET, in Buenos Aires, Argentina from 2011 to 2022 (age of onset 20 to 50 years old). Most of the cases were sporadic (90/100), while a few cases reported clinical familial history of vision loss (10/100).

The inclusion criterion was sudden onset painless visual loss in a patient in whom other more frequent conditions (such as ischemic events or rheumatologic disorders). All the patients referred to the molecular study were previously clinically reviewed by an ophthalmologist and a neuro-ophthalmologist. They underwent Humphrey visual field (HVF) testing the reduction of visual acuity (VA) associated with impaired colour perception and central scotoma at visual field (VF); dilated fundoscopy to reveal temporal pallor of the optic nerve; neurological examination of motor and sensory function, muscle bulk, tone, and gait; magnetic resonance imaging (MRI) to show optic nerve appearance. Gathering all the information together, they are referred to the genetic service for their molecular study. The protocol was approved by the institutional ethics committee “Fundación para la lucha contra enfermedades neurológicas de la infancia (FLENI)” and was conducted according to the Helsinki Declaration. Written informed consent was obtained for all study subjects prior to molecular studies.

### Samples

Whole blood was drawn by venipuncture with 5% ethylene-diamine tetraacetic acid (EDTA) as anticoagulant for all study subjects. Genomic DNA was isolated using the cetyl-trimethyl-ammonium bromide method [29, 30]. DNA concentration and quality were measured by absorbance at 260 nm and by the A260 nm/A280 nm and A260 nm/A230 nm ratios, respectively (NanoDropTM) (Thermo Fisher Scientific, Wilmington, NC, USA), and DNA quality was confirmed by 1% agarose gel electrophoresis. All samples were stored at −20 °C.

### Genetic variant screening

Polymerase chain reaction (PCR) and Sanger Sequencing were performed targeting the entire sequence of the genes *MT-ND*1, *MT-ND*4 and *MT-ND*6 (primers upon request). The PCR reaction mix contained: 200 uM dNTPs, 1.5 mM MgCl2, 20 mM Tris ClH (pH 8), 50 mM KCl and 1 U Taq polymerase (Invitrogen, Thermo Fisher Scientific,California, USA) in a final volume of 25 ul. A ProFlex thermal cycler (Thermo Fisher Scientific, Wilmington, NC, USA) was used. The PCR protocol consisted of: 30 cycles with annealing at 53°-58°C for 30s, extension at 72°C for 1 min, denaturation at 94° for 40 s, with an initial denaturation step at 95 for 5 min and a final extension step at 72 for 10 min.

Bidirectional DNA sequencing was performed on an automatic sequencer (3730xl DNA Analyzer, Applied Biosystems, Foster City, CA, USA). Sequence results were analyzed with CodonCodeAligner program [31, 32] and BLAST NCBI interface (Basic local alignment search tool) [33] using the consensus sequence of mitochondrial genome (GeneBank NC_012920.1). Clinical Interpretation of Sequence Variants was based on MITOMAP curated database classification [https://www.mitomap.org/MITOMAP]. When needed, further analysis was achieved following the latest specifications of the American College of Medical Genetics and Genomics and the Association for Molecular Pathology (ACMG/AMP) standards and guidelines for mitochondrial DNA variant interpretation [34]. HmtVar and APOGEE were used in addition to literature search [35, 36]. The final criteria score was manually assigned through the InterVar bioinformatic software [37].

## Results

**Fig 1:**
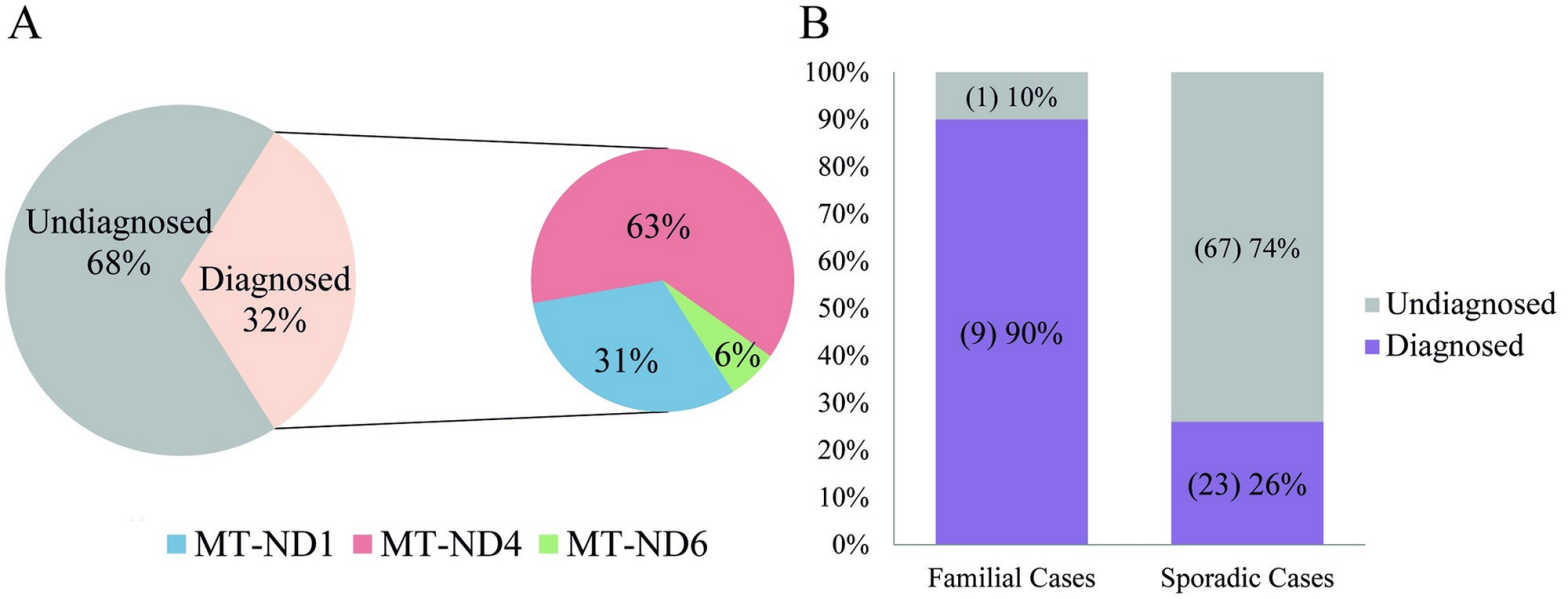
Identification of disease-causing variants. **A,** General diagnostic rate and frequency of genes harboring pathogenic variants in diagnosed patients. **B,** Diagnostic rate in family and sporadic cases.

A total of 100 patients with suspected LHON were tested for mutations in *MT-ND*1, *MT-ND*4 and *MT-ND*6 by Sanger Sequencing. In 32% of the cases, a molecular confirmation of the diagnosis was established, due to the identification of disease-causing variants (32/100). Among these, mutations in *MT-ND*4 were responsible for 20/32 of the cases, accounting for 63% of the disease-causing variants detected, followed by *MT-ND*1 variants 10/32 (31%) and *MT-ND*6 variants 2/32 (6%). Moreover, disease-causing variants were identified in almost all the familial cases (9/10), whereas they were found in 26% of sporadic ones (23/90) (Fig 1).

Overall, 54 different genetic variants were detected in 82% of the patients screened (82/100). Among this group, 5 sequence variants which were detected in 32 patients were classified as disease-causing in MitoMap. The most frequent pathogenic variant detected in our cohort was m.11778G>A p.Arg340His in MT-ND4 (17/100), representing 17% of tested patients, followed by m.3460G>A p.Ala52Thr in MT-ND1 (9%; 9/100). Additionally to the frequently reported variants, m.11253T>C p.Ile165Thr in *MT-ND*4 and m.3395A>G p.Tyr30Cys in *MT-ND*1 were identified in 3 and 1 patients, respectively (Table 1).

**Table 1:** Disease-causing variants identified in the Argentinean cohort.

Gene	Genetic Variant	Protein Change	Number of affected individuals (percentage)	GB Frequency
*MT-ND*1	m.3460G>A	p.Ala52Thr	9 (9%) *	0.060%
	m.3395A>G	p.Tyr30Cys	1 (1%) #	0.049%
*MT-ND*4	m.11253T>C	p.Ile165Thr	3 (3%)	0.503%
	m.11778G>A	p.Arg340His	17 (17%)	0%
*MT-ND*6	m.14484 T>C	p.Met64Val	2 (2%)	0.122%

GenBank Frequency Information (GB) was obtained from data in Mitomap: http://www.mitomap.org/MITOMAP/GBFreqInfo). * In two patients (one isolated and one familial case) variant m.3460G>A p.Ala52Thr was detected in heteroplasmic state. # variant m.3395A>G p.Tyr30Cys was detected in a patient with LHON, deafness and parkinsonism. The rest of the variants have been detected in homoplasmic state.

In addition to the three variants frequently reported worldwide, two other rare mutations were detected: m.11253T>C p.Ile165Thr in *MT-ND*6 and m.3395A>G p.Tyr30Cys in *MT-ND*1. The m.11253T>C in *MT-ND*6 was identified in three sporadic male cases. It was classified as Benign in ClinVar (Accession Number: VCV000065509.4, 2019) and notably as Disease Causing in MitoMap database. Considering the recent ACMG guidelines for analyzing mitochondrial variants, we performed a manual variant curation. The variant frequency is reported between 0.506%-0.948% depending on the database which meets BS1 criteria. *In silico* analysis, using the APOGEE algorithm, resulted in pathogenic prediction, which is enough to apply PP3 criteria. This variant has been identified in 10 different LHON probands, and therefore PS4_Moderate is applied. Considering all the evidence, BS1, PP3 and PS4_moderate, the variant m.11253T>C is re-interpreted as uncertain significance (Table 2). However, we consider the parameter PS4_moderate excessively strict, since according to specific standard guides for mitochondrial variants, a total of 16 probands carrying the same mutation is needed to apply PS4 (strong) and therefore, we suggest considering the m.11253T>C variant as VUS_likely pathogenic to be further studied worldwide.

**Table 2:** Summary of rules applied m.11253T>C p.Ile165Thr in *MT-ND*6.

Rules Applied	Explanation	Reference/ Evidence
BS1	Frequency (0.506% in GenBank, 0.673% in GnomAD 3.1 and 0.948% in Helix)	[www.mitomap.org]
		[https://gnomad.broadinstitute.org/]
		[https://www.helix.com/]
PP3	APOGEE score>0,5 (0.53)	[https://mitimpact.css-mendel.it/]
PS4_Moderate	Variant present in 10 unrelated probands.	[2, 38–41]

Regarding, the variant m.3395A>G, p.Tyr30Cys, was identified in one of the isolated cases analyzed. The patient was diagnosed with loss of vision, moderate deafness (70db and 50db in left and right ear respectively), paresthesias, migraines and parkinsonism. Symptoms started 6 years ago with loss of hearing, and progressed a year after that with neurological signs. She was positive for HSV, CMV and Toxoplasma IgG and received treatment with corticosteroids. Imaging studies were normal, but showed alterations in visual evoked potentials, with compromise of the optic nerve. Of note, contradictory interpretation of this variant has been reported in databases, since it is classified as Disease Causing in MITOMAP, but as Likely Benign in ClinVar (Accession Number: VCV000692350). In order to clarify the variant classification, a detailed analysis of the publications and reports was done and a manual curation was performed in accordance with the recommendations of the ACMG/AMP in the context of mitochondrial DNA variant analysis. After the curation process, criteria applied were: BS1, PP3, PS3_Supp, PP4, PM5, PS4_Moderate and the final classification was Likely Pathogenic (Table 3).

**Table 3:** Summary of rules applied for m.3395A>G p.(Tyr30Cys) variant curation in *MT-ND*1.

Rules Applied	Explanation	Reference/ Evidence
BS1	Frequency of 0,49%	[www.mitomap.org]
PP3	APOGEE score>0,5 (0.62)	[https://mitimpact.css-mendel.it/]
PS3_Supp	Functional Data decrease of 22% for the cybrid cell lines harboring mutation	[42]
PP4	Decrease Electron transport chain enzyme activity (patient’s phenotype highly specific for a disease)	[43, 44]
PM5	Previous pathogenic missense change in the same amino acid residue [p.(Y30H)]	[45]
PS4_Moderate	Variant present in 4 unrelated probands.	[43, 44, 46], This Report

Ten familial cases were studied, and 9/10 were diagnosed with pathogenic variants in *MT-ND*1 and *MT-ND*4 genes. The undiagnosed patient and her son suffered from loss of visual acuity since childhood, however, since results were negative and mendelian inheritance could not be ruled out, she was referred for further genetic tests. Three familial cases are described below.

**Fig 2:**
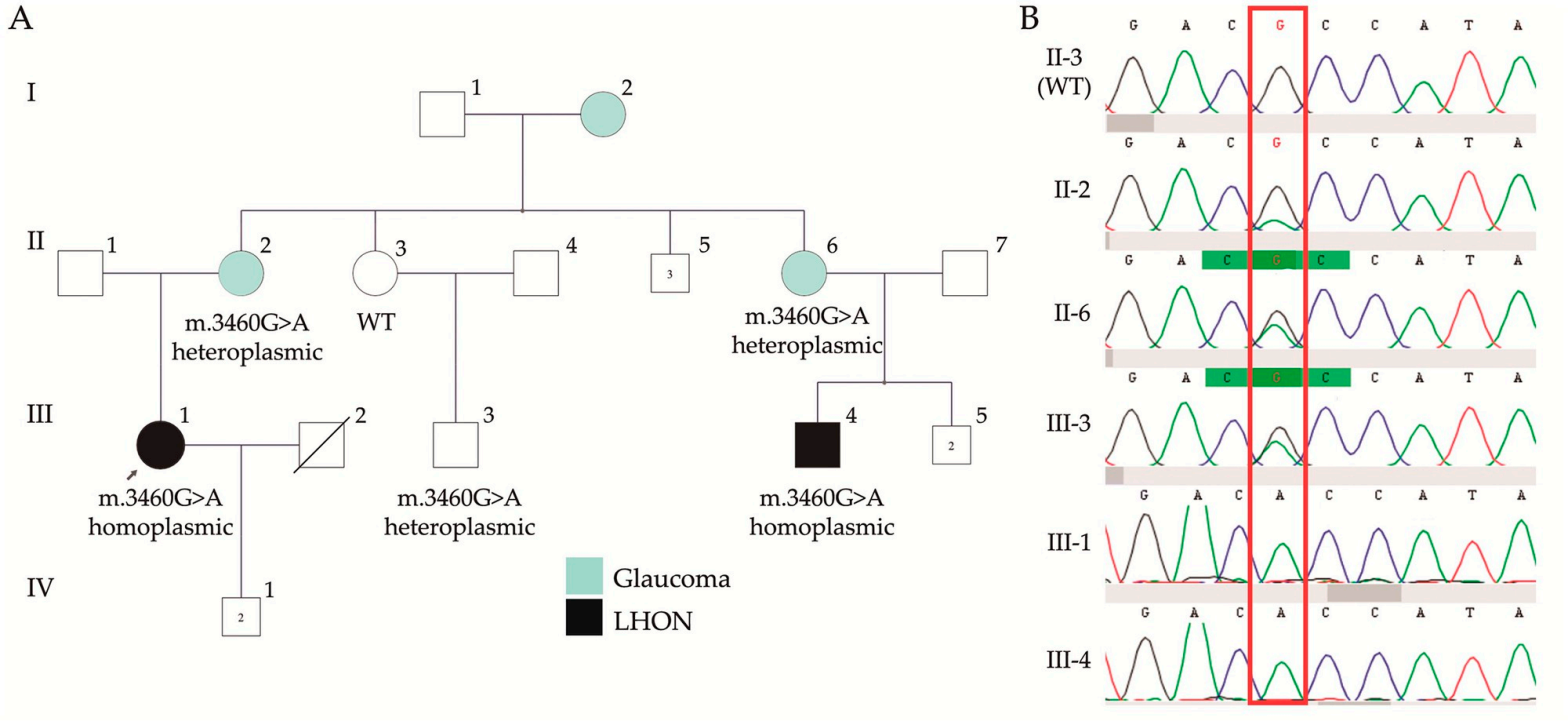
Family with variant m.3460G>A p.Ala52Thr in the *MT-ND*1 gene. A, The variant was detected in both affected cousins III1 and III4. The analysis in the family revealed heteroplasmic segregation in II2, II6 and III3, unaffected for visual loss but who suffered glaucoma. The genetic variant was not detected by Sanger sequencing in the obligated carrier II3. B, Partial sequence chromatograms of ND1 gene from studied individuals of the family. Red square indicates the location of the base changes at positions 3460.

**Fig 3:**
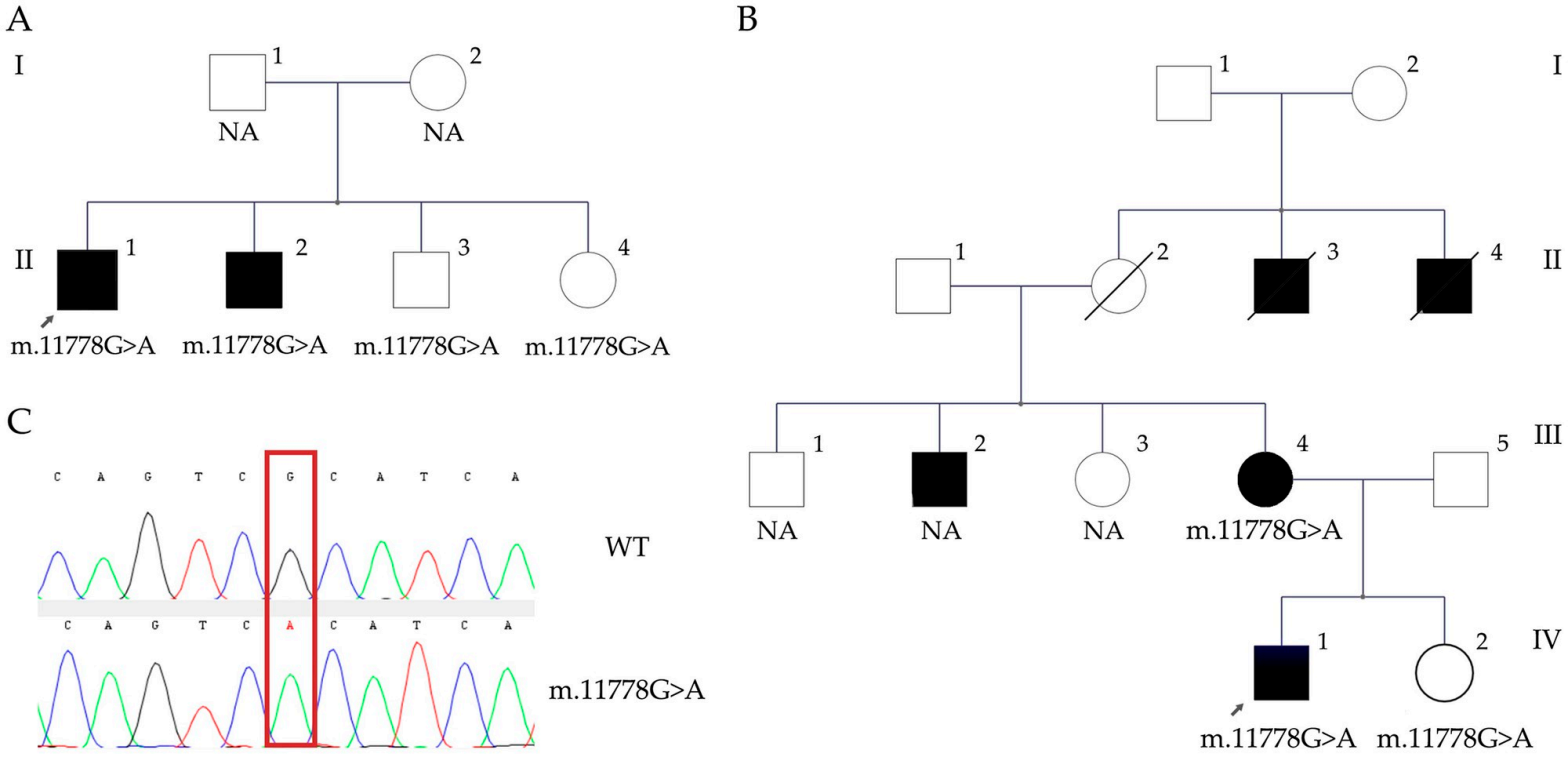
Families with variant m.11778G>A, p.Arg340His, in the *MT-ND*4 gene. A, Four members of a family resulted positive for pathogenic variants in *MT-ND*4 gene, but only II1 and II2 had developed visual loss at the time of the study. B, A 40 year old patient with diabetes, history of leukemia and sudden bilateral loss of visual acuity (IV1). His non-smoker mother suffered diabetes, polyneuropathy, migraine and visual loss. The sister remains asymptomatic (IV2). C, partial sequence chromatograms of ND4 gene. Red square indicates the location of the base changes at position 11778.

Case 1:
  A family with two affected cousins and several presumably unaffected members was screened for variants in *MT-ND*1, *MT-ND*4 and *MT-ND*6 genes. After genetic testing, the frequent variant m.3460G>A p.Ala52Thr in MT-*ND1* was detected in both affected cousins (III1 and III4). Notably, the analysis in the family revealed heteroplasmic segregation of the mtDNA variant in three other unaffected members (II2, II6 and III3). The genetic variant was not detected in the obligated carrier II3, who may be clinically unaffected but who must carry the genetic variant m.3460G>A based on pedigree (Fig 2A and 2B), presumably located in tissues other than peripheral blood (including gonads). After testing, all heteroplasmic carriers of the family were clinically evaluated, none of them showed visual failure, loss of acuity, central scotoma, optic atrophy or nystagmus. Nevertheless, all of the patients with heteroplasmic status suffered glaucoma.
Case 2:
  A family with two affected and two unaffected brothers was screened for variants in *MT-ND*1, *MT-ND*4 and *MT-ND*6 genes. After genetic testing, the frequent variant m.11778G>A p.Arg340His in *MT-ND4* gene was detected in all four members, but only II1 and II2 (in their twenties) had developed visual loss at the time of the study (Fig 3A). II1 was under idebenone treatment (45mg / 5 times a day), with a slight improvement of the visual field. Since unaffected carriers in the family are still around 18–20 years old, strict ophthalmologic follow-up was recommended, as well as avoiding the consumption of tobacco and alcohol.
Case 3:
  A 40-year old patient was evaluated for sudden bilateral loss of visual acuity. He was a social smoker and had been diagnosed with type 2 diabetes five months before consultation; he also had a history of leukemia. Ophthalmological tests revealed optic nerve hypofluorescence and alterations on the pigmentary epithelium of the retina, with papillary atrophy, and a normal brain CT scan. His non-smoker 59 year-old mother (III4) suffered an intense migraine and syncope with a subsequent unilateral visual loss and a compromise of the contralateral vision a week later. Diabetes and polyneuropathy, with insulin and pregabalin requirements, were also diagnosed. Both the patient and his mother harbor the variant m.11778G>A p.Arg340His in the *MT-ND4* gene (Fig 3B). Other members of the family were not available, but several were suggested to be affected (II3, II4, III2), and one 40 year-old healthy available sister harbored the m.11778G>A variant as well.

In addition to the pathogenic and likely pathogenic variants identified, other 49 mitochondrial genetic variants reported as benign in MITOMAP were detected in homoplasmic state in our cohort of 100 patients. The m.11719G>A variant in MT-*ND4* gene (p.Gly320Gly) was the most frequent one, representing 57% of the patients tested. It was followed by m.3547A>G p.Ile81Val (15%) marker for the B2 haplogroup, m.14318T>C p.Asn119Ser (14%) and m.3552T>A p.Ala82Ala (12%) markers for C haplogroup; m.4248T>C p.Ile314Ile (7%) indicator for the AE; and m.4216T>C p.Tyr304His (6%) for the JT haplogroup. The frequency of the other benign variants is 1–3% (Table 4).

**Table 4:** Genetic variants reported as benign in MITOMAP detected in 100 patients tested.

Gene	Genetic Variant	Protein Change	Number of patients
*MT-ND*1	m.3308T>C	p.Met1Thr	1
	m.3316G>A	p.Ala4Thr	1
	m.3372T>C	p.Leu22Leu	1
	m.3397A>G	p.Met31Val	2
	m.3438G>A	p.Gly44Gly	3
	m.3447A>G	p.Gln47Gln	1
	m.3480A>G	p.Lys58Lys	1
	m.3483G>A	p.Glu59Glu	1
	m.3504T>C	p.Ser66Ser	1
	m.3535T>C	p.Leu77Leu	1
	m.3547A>G	p.Ile81Val	15
	m.3548T>C	p.Ile81Thr	1
	m.3552T>A	p.Ala82Ala	12
	m.3591G>A	p.Leu95Leu	1
	m.3594C>T	p.Val96Val	1
	m.3666G>A	p.Gly120Gly	1
	m.3693G>A	p.Leu129Leu	1
	m.3746C>T	p.Ala147Val	3
	m.3808A>G	p.Thr168Ala	1
	m.3915G>A	p.Gly203Gly	2
	m.3918G>A	p.Glu204Glu	1
	m.3992C>T	p.Thr229Met	1
	m.3999T>C	p.Ile231Ile	1
	m.4024A>G	p.Thr240Ala	1
	m.4092G>A	p.Lys262Lys	2
	m.4104A>G	p.Leu266Leu	2
	m.4216T>C	p.Tyr304His	6
	m.4248T>C	p.Ile314Ile	7
	m.4259C>T	p.Thr318Ile	1
*MT-ND*4	m.11251A>G	p.Leu164Leu	3
	m.11299T>C	p.Thr180Thr	1
	m.11377G>A	p.Lys206Lys	1
	m.11467A>G	p.Leu236Leu	1
	m.11611G>A	p.Ser284Ser	2
	m.11776T>C	p.Ser339Ser	2
	m.11719G>A	p.Gly320Gly	56
*MT-ND*6	m.14305G>A	p.Ser123Ser	1
	m.14318T>C	p.Asn119Ser	14
	m.14359C>T	p.Trp105Trp	1
	m.14364G>A	p.Leu104Leu	1
	m.14410G>A	p.Val88Val	1
	m.14461T>C	p.Trp71Trp	1
	m.14527A>G	p.Gly49Gly	1
	m.14560G>A	p.Val38Val	1
	m.14566A>G	p.Gly36Gly	1
	m.14561A>G	p.Val38Ala	1
	m.14582A>G	p.Val31Ala	1
	m.14587A>G	p.Gly29Gly	4
	m.14634T>C	p.Met14Val	1

## Discussion

This study shows, for the first time, the detection and analysis of mtDNA variants in patients with a presumptive clinical diagnosis of LHON in Argentina. According to the physicians referral, all cases presented phenotypes compatible with the pathology. However, applying standard Sanger sequencing, it was possible to achieve a genetic diagnosis only in 32% of the cases, compared to the 90–95% reported in other countries [3]. This result suggests that under the present conditions, Sanger sequencing is only an effective first approach, especially for centers from low-middle income countries, leaving NGS studies for those patients with negative or inconclusive results. The low incidence of solved cases in our cohort might result from poor complementary examinations performed in order to rule out a clinical differential diagnosis of other hereditary optic neuropathies as dominant optic atrophies autosomal inherited, prior to the molecular diagnosis. Thus, the reported 32% of diagnostic range of our study vs the 90% reported worldwide most likely indicates that only one third of the suspected LHON cases referred to molecular diagnosis analysed are indeed “classical” LHON patients.

The five disease-causing mtDNA variants in the present study are in the ND subunits of complex I. Moreover 87,5% of patients with a resolved molecular diagnosis exhibited the G3460A+G11778A+T14484C variants in *ND*1-*ND*4-*ND*6. This reflects what has been reported in the literature for other populations, where 90% of LHON cases are due to those most frequent reported mutations (Mitomap database: https://www.mitomap.org). However, it should be noted that nuclear genes such as *DNAJC*30, *NDUFS*2 and *MCAT*, reported in some cases of LHON patients [47, 48], were not tested in our present study and could account for some of the present unsolved LHON patients. Moreover, variants in nuclear genes *OPA1*, *OPA3*, *TYMP* and *POLG* have been reported in patients with other optic atrophies with phenotypes that could be similar to LHON [49–51]. Further molecular testing of these genes in our cohort, might guide future clinical diagnosis in Argentina ophthalmology patients to dissect overlapping symptomatology with other optic atrophies. Moreover, further molecular testing, including NGS, might resolved the low incidence of detection in our cohort. On the other hand, a more extensive genetic analysis, can be extremely useful to analyze and interpret the diversity of variants in our population (e.g., a high number of *DNAJC*30 cases could suggest an autosomal inheritance as reported in European patients and a common founder effect as reported in Russian population [52].

All of the variants detected are present in diverse genomic databases (Mitomap, Ensembl, and Genome Browser), and other 49 benign variants reported in the mitomap database were detected in the mtDNA of patients. The most frequent benign variants detected (excluding the m.11719G>A) were the m.3547A>G marker for B2 haplogroup (15%) and the m.14318T>C and m.3552T>C diagnostic for C haplogroup (14% and 12% respectively). They are followed with low frequencies by m.4248T>C from haplogroups AE (7%) and m.4216T>C marker for JT (6%). Haplogroups A, B2, C are associated to Native Americans [53, 54]. This is in concordance with previous reports from Argentina which showcased that the 4 major haplogroups in our country are A2, B2, C1, and D1 and its particular frequencies depend on the Argentinean region studied [55–57]. Haplogroups JT correspond to the European population [53]. These outcomes demonstrate the mingling of populations as a result of the human colonization of the Americas and the construction of the Argentinean Nation [55].

To the best of our knowledge, only 1 patient with LHON from our country has been reported before, in a publication studying 27 patients with diverse pediatric mtDNA disorders [58], making the results of these 100 patients in our country the first report showing the prevalence of mutations for LHON patients in Argentina, and which includes mostly adult patients. The diagnostic algorithm, including the study of point mtDNA variants, was efficient and advantageous for this study, and even when the higher diagnostic yield of running whole mtDNA sequence analysis is proven, this test limited to frequent mutations resulted in a beneficial cost-effective study for routine analysis of patients in our country.

The clinical characteristics of LHON overlap in part with those of several other optic neuropathy etiologies, including monogenic nuclear optic nerve atrophies, so molecular testing is a helpful tool for establishing the diagnosis. In our cohort, routine mtDNA screening covering primary LHON mtDNA mutations is successful in 90% of families with multigenerational visual loss compatible with maternal inheritance, in accordance with several reports worldwide [7]. In this regard, the most frequent disease causing variants are m.11778G>A in *MT-ND*4, accounting for 60%-70% of the cases from Northern European and Asian populations [7, 59, 60]; m.14484T>C in *MT-ND*6 in 14%, most common among French Canadians [61, 62]; and m.3460G>A in *MT-ND*1 (13% of all cases and accounting for about 35% of European LHON) [63]. Ten familial cases were studied in order to identify causative mutations in ND genes. After scrutiny, frequent mutations in *MT-ND*1 and *MT-ND*4 were found in 9/10 of the cases, and they segregated in all of the affected members of the families. Nevertheless, it appeared that m.3460A>G in *MT-ND*1 was found in heteroplasmy in three samples (II2, II6 and III3 in family 1A). Members of the family with variants in homoplasmic state (III1 and III4) have a habitual loss of visual acuity and optic neuropathy, whereas in heteroplasmic carriers only glaucoma was diagnosed with no other affection and no loss of vision. Previous reports described similar clinical manifestations between patients with glaucoma and LHON disease such as enlarged cup-to-disc ratio, optic nerve head cupping and visual field damage [64–67]. Moreover, there are other case reports where probands with disease-causing variants in MT-ND4 develop glaucoma, suggesting that high intraocular pressure could have some effect on the oxidative stress caused by the mtDNA mutation participating in the retinal damage [68–70].

Although m.3460A>G in *MT-ND*1 is linked with LHON and other syndromes, none of the probands in this family developed so far any other sign other than visual ones. The genetic variant was not detected in the obligated carrier II3, who may be clinically unaffected but should have received and transmitted the genetic variant m.3460G>A based on analysis of the family history. The heteroplasmic genetic variant is supposed to be present in at least a level above 30%-50% in blood to be detected by Sanger sequencing [71]. As the proportion of affected mitochondria may vary among tissues and over time when variants are in heteroplasmic state, it might be necessary to test other affected tissues in order to establish a proper diagnosis, improving the reliability and sensitivity of mtDNA tests (such as Next Generation Sequencing). Therefore, as no NGS tests were available for studying the family in the present work, these test results should be interpreted with caution.

Regarding rare genetic variants, we detected the m.11253T>C p.Ile165Thr in *MT-ND*6 in three unrelated patients. Since there was a conflicting interpretation of its pathogenicity (Benign in ClinVar and Disease Causing in MitoMap), we carried out a manual curation. Considering all the gathered evidence (frequency, variant detection in unrelated probands and *in silico* analysis) the variant met criteria to be classified as uncertain significance. However, we consider that 10 unrelated probands is solid evidence enough respecting variant detection in LHON patients. Besides, a previous report functionally studied the effect of m.11253T>C simultaneously with m.11778G>A or m.14484T>C, which revealed no differences in mitochondrial function, demonstrating the absence of a synergistic effect in this model system [72]. A study of the isolated m.11253T>C mutation as well as new reports of LHON patients carrying this variant, would provide valuable evidence to unequivocally classify it as Likely Pathogenic since PS3_Supporting and PS4_Strong criteria could be respectively applied.

Here, we described one patient carrying a homoplasmic mtDNA mutation in the gene coding for subunit ND1 of complex I: m.3395A>G p.Tyr30Cys. The patient suffered from LHON, deafness and parkinsonism. The same mutation has been reported previously in five patients, from three unrelated families, suffering from isolated non-syndromic deafness to multisystemic disorder. The mutation is non-synonymous and affects a highly conserved amino acid (conservation index between 89 and 94%) in position 30. Moreover, it was proven to be pathogenic in cytoplasmic hybrid cells (cybrid) by affecting the quantity of the protein in the functional experiment and acting as a limiting factor of complex I assembly [42]. This variant was curated following the recent ACMG specific guidelines for analyzing mitochondrial variants, since discrepancies were found in its classification between MitoMap (Disease causing) and the report uploaded to ClinVar (Likely Benign). Available information was scrutinized considering that conflicting interpretation of variants has been reported to lead to a negative impact in patient health and their precise assessment [73, 74]. Although the frequency of the variant is higher than expected for the disease (and therefore the BS1 criteria was applied), the evidence of pathogenicity was sufficient to classify the variant as Likely Pathogenic according to specific mitochondrial ACMG guidelines. Gathered data regarding functional experiments reported by [42] proved its pathogenic impact. Patient’s phenotype was highly specific for the disease, and a previous change in the same amino acid position and computational predictions of disease were also available. The information collected for its original evaluation was insufficient at the time; since the variant had been submitted to ClinVar in October 2019 and functional evidence of its pathogenicity was published in April 2020 as well as specifications of ACMG for mitochondrial variants in December 2020. This showcased the importance of re-evaluating uncertain or conflicting variants after a period of time under the light of new available evidence, as well as the need for specific criteria to analyze genetic variants in the context of mitochondrial diseases. Other patients with this mutation present deafness, exercise intolerance and a patient developed a cerebellar syndrome. Some authors have proposed that the impact of this mutation may be conditioned by the haplogroup of the carrier to reveal a possible influence of the mtDNA genetic background on the phenotypic expression of the mutation but not tested in the present study [15]. Pathogenic variants on the J background are associated with an increased penetrance of the disease [75–78]. In this regard, the variant m.4216T>C that it was detected six times, was the only variant related to the JT haplogroup in our studied cohort, resulting in an infrequent haplogroup in our samples. Moreover, it was detected only in one diagnosed patient with pathogenic variant m.14484T>C in the MT-ND6 gene (S1 Table). As it was expected, we could determine that B2 and C haplogroups were more prevalent than J in our cohort considering the NativeAmerican background in our country. This finding results in valuable information for genetic counseling, allowing to assess a lower penetrance of this complex disease in our cohort. Studying the patients mitogenome could provide accurate evidence on haplogroup prevalence.

LHON is characterized by rapidly progressive, though painless, loss of vision with central visual scotoma. This presentation is shared with toxic and nutritional optic neuropathies (TNONs), with the exception that, in the latter, visual loss is usually experienced simultaneously in both eyes. A strong and consistent association was reported between visual loss in LHON and smoking tobacco or alcohol intake, independently of gender [79], so these toxic substances are pointed out as possible triggers for the symptoms. Thus this information is shared to all patients, upon diagnosis, and exposure to these toxics is discouraged. The primary LHON mutation is a prerequisite for visual loss, but secondary factors are clearly modulating the risk of visual loss precipitating optic nerve dysfunction. Other epigenetics factors, exogenous influences, light exposure and pharmacological agents might have putative mitochondrial toxic effects as well, modulating the phenotypic expression of LHON [5, 79, 80]. In this regard, oxidative stress modulation has been at the center of treatment strategies, increasing mitochondrial respiration and reducing ROS produced. The most advanced yet invasive forms of therapy involve gene therapy (GT) strategies delivering a gene to the nucleus of the RGC and leading to the production of a redirected to the mitochondria protein. Because the *MT-ND*4 mutation is the most common cause of LHON, GT efforts tend to focus on it, therefore making genetic diagnosis essential to access to the appropriate treatment and to help in the decision making process for patient clinical management [19, 25, 26]. Sudden visual impairment in LHON is experienced as dramatic in the life of patients, and the inability to cope with simple, everyday tasks is devastating for most of them, especially when absence of family history provides them with no warning of such an event. This unforeseen change of social life often comes with high cost in psychological structure of patients, and genetic diagnosis and counseling help patients to adapt to the physiological and familial implications of LHON, and may even allow them to reduce the risk of recurrence of the condition in other family members. Management of patients and families must be multidisciplinary and lifelong, and includes applying prevention strategies and rehabilitation, which currently remain the only ways to improve the patients’ quality of life.

## Supporting information

S1 Table (XLSX)
